# Population Structure and Evidence for Both Clonality and Recombination among Brazilian Strains of the Subgenus *Leishmania* (*Viannia*)

**DOI:** 10.1371/journal.pntd.0002490

**Published:** 2013-10-31

**Authors:** Katrin Kuhls, Elisa Cupolillo, Soraia O. Silva, Carola Schweynoch, Mariana Côrtes Boité, Maria N. Mello, Isabel Mauricio, Michael Miles, Thierry Wirth, Gabriele Schönian

**Affiliations:** 1 Institut für Mikrobiologie und Hygiene, Charité Universitätsmedizin Berlin, Berlin, Germany; 2 Laboratório de Pesquisa em Leishmaniose, Instituto Oswaldo Cruz - Fiocruz, Rio de Janeiro, Brazil; 3 Universidade Federal de Minas Gerais, Belo Horizonte, Brazil; 4 Instituto de Higiene e Medicina Tropical, Lisboa, Portugal; 5 Department of Pathogen Molecular Biology, Faculty of Infectious and Tropical Diseases, London School of Hygiene and Tropical Medicine, London, United Kingdom; 6 Ecole Pratique des Hautes Etudes, Muséum National d'Histoire Naturelle, Département de Systématique et Évolution, UMR-CNRS 7205, Paris, France; University of Montpellier, France

## Abstract

***Background/Objectives:*** Parasites of the subgenus *Leishmania (Viannia)* cause varying clinical symptoms ranging from cutaneous leishmaniases (CL) with single or few lesions, disseminated CL (DL) with multiple lesions to disfiguring forms of mucocutaneous leishmaniasis (MCL). In this population genetics study, 37 strains of *L. (V.) guyanensis*, 63 of *L. (V.) braziliensis*, four of *L. (V.) shawi*, six of *L. (V.) lainsoni*, seven of *L. (V.) naiffi*, one each of *L. (V.) utingensis* and *L. (V.) lindenbergi*, and one *L. (V.) lainsoni*/*L. naiffi* hybrid from different endemic foci in Brazil were examined for variation at 15 hyper-variable microsatellite markers.

***Methodology/Principal findings: ***The multilocus microsatellite profiles obtained for the 120 strains were analysed using both model- and distance-based methods. Significant genetic diversity was observed for all *L. (Viannia)* strains studied. The two cluster analysis approaches identified two principal genetic groups or populations, one consisting of strains of *L. (V.) guyanensis* from the Amazon region and the other of strains of *L. (V.) braziliensis* isolated along the Atlantic coast of Brazil. A third group comprised a heterogeneous assembly of species, including other strains of *L. braziliensis* isolated from the north of Brazil, which were extremely polymorphic. The latter strains seemed to be more closely related to those of *L. (V.) shawi*, *L. (V.) naiffi*, and *L. (V.) lainsoni*, also isolated in northern Brazilian foci. The MLMT approach identified an epidemic clone consisting of 13 strains of *L. braziliensis* from Minas Gerais, but evidence for recombination was obtained for the populations of *L. (V.) braziliensis* from the Atlantic coast and for *L. (V.) guyanensis*.

***Conclusions/Significance:*** Different levels of recombination versus clonality seem to occur within the subgenus *L.* (*Viannia*). Though clearly departing from panmixia, sporadic, but long-term sustained recombination might explain the tremendous genetic diversity and limited population structure found for such *L.* (*Viannia*) strains.

## Author Summary

Cutaneous leishmaniasis (CL) constitutes a significant public health problem in all federal states of Brazil. Most cases are caused by parasites of the subgenus *Leishmania (Viannia)* which can cause a variety of clinical symptoms ranging from single or few lesions, disseminated CL with multiple lesions, to disfiguring forms of mucocutaneous leishmaniasis. This study has used a multilocus microsatellite typing approach for exploring the genetic diversity and population structure among 120 strains representing different subgenus *L. (Viannia)* species and different Brazilian CL foci. Genetic diversity within the subgenus was much higher than expected, especially within *L. (V.) braziliensis*, *L. (V.) shawi*, *L. (V.) naiffi*, and *L. (V.) lainsoni* which were all from the north of Brazil. These strains could not be assigned to well-defined populations, but presented a rather loosely associated group. Strains of *L. (V.) braziliensis* isolated along the Atlantic coast of Brazil and strains of *L. (V.) guyanensis* formed, however, two clearly separated populations exhibiting remarkable levels of sexual exchange. The latter finding is in contrast to previous studies suggesting clonal modes of propagation or inbreeding for natural populations of *Leishmania* parasites and might explain the genetic heterogeneity and limited population structure for Brazilian strains of subgenus *L.* (*Viannia*) observed in this study.

## Introduction

The species of the subgenus *Leishmania (Viannia)* Lainson and Shaw, 1987, are exclusively endemic in the New World (NW) and infections of humans with these protozoan parasites constitute a significant public health problem in at least 18 countries of Latin America [1]. Subgenus *L. (Viannia)* parasites are capable of causing a variety of clinical symptoms ranging from cutaneous leishmaniasis (CL) with single or few lesions that may heal spontaneously, disseminated CL (DL) with multiple lesions, to disfiguring forms of mucocutaneous leishmaniasis (MCL) that may occur concomitantly or after remission of CL [2]. The outcome of human infections by *Leishmania* parasites is thought to be influenced by the immune status of the host and virulence of the infecting parasite [3]. At present, multilocus enzyme electrophoresis (MLEE) is the reference technique for the identification of *Leishmania* and was employed in most of the classification schemes, although MLEE is likely to be partially superseded by multilocus sequence typing (MLST). The application of numerical taxonomy and cladistic techniques to electrophoretic data has resulted in the identification of two species complexes in the subgenus *L. (Viannia)*, namely the *L. (V.) braziliensis* complex comprising *L. (V.) braziliensis* and *L. (V.) peruviana*, and the *L. (V.) guyanensis* complex comprising *L. (V.) guyanensis*, *L. (V.) panamensis* and *L. (V.) shawi*, and of at least four single species, *L. (V.) lainsoni*, *L. (V.) naiffi*, *L. (V.) lindenbergi* and *L. (V.) utingensis* (for review see [4]). This classification has been largely supported by a recent molecular study comparing *hsp*70 sequences of different *Leishmania* species [5].

In Brazil, CL is endemic in all federal states and an annual mean of 27,250 CL cases has been registered from 1990–2010 (http://portal.saude.gov.br/portal/saude/profissional/area.cfm?id_area=1560). The disease is caused by six species of the subgenus *L.* (*Viannia*), *L. (V.) braziliensis*, *L. (V.) guyanensis*, *L. (V.) shawi*, *L. (V.) lainsoni*, *L. (V.) naiffi* and *L. (V.) lindenbergi* plus one species of the subgenus *L. (Leishmania)*, *L. (L.) amazonensis*. Around 6–7% of the CL patients will develop symptoms of MCL [6] mainly after infection with *L. (V.) braziliensis* and, to a lesser extent, also with *L. (V.) guyanensis* [7], [8]. So far, DL has been also mainly associated with *L. (V.) braziliensis*, and only sporadic cases with *L. (V.) guyanensis* [1]. Severe anergic diffuse cutaneous leishmaniasis (DCL) may be a long term sequel in a minority of *L. (L.) amazonensis* infections [1].

Transmission of species of the subgenus *L.* (*Viannia*) involves different species of phlebotomine sand flies and a wide variety of wild and domestic animals have been implicated as reservoir hosts. Some species have a more restricted transmission cycle, whereas others are more complex with several different vectors and hosts in different ecological and geographical regions [1], [3]. Sympatry of different subgenus *L. (Viannia)* species has been reported particularly in the Amazon region [9] where separate epidemiological patterns have been described, involving different sand fly species [10]. There is also increasing evidence that pathogenic *Leishmania* strains can be maintained in both sylvatic cycles, involving wild animals and sylvatic sand flies, and urban cycles involving domestic animals and peridomestic sand flies [11]. Subgenus *L.* (*Viannia*) parasites are characterized by tremendous genetic diversity and the described species vary enormously in their degree of such diversity [12], [13]. Intra-specific polymorphisms are, for example, very frequent in *L. (V.) braziliensis* and *L. (V) naiffi*, and it has been suggested that the genetic diversity of the parasites is most probably related to the sand fly vector(s) and/or animal reservoir(s) involved in the transmission cycles. On the other hand, *L. (V.) guyanensis*, particularly strains circulating in the Brazilian Amazon region, and *L. (V.) shawi* have been found to be rather homogenous by MLEE and ITS-RFLP typing [12], [13].

Multilocus microsatellite typing (MLMT) is currently the method of choice for molecular epidemiological and population genetic studies of different species of *Leishmania* (reviewed in [14]). It combines the advantages of co-dominance and higher discriminatory power when compared to MLEE, RAPD and the PCR-RFLP approaches used in many studies. Different sets of microsatellite markers have been designed and successfully applied for discriminating strains of subgenus *L. (Viannia)* with special emphasis on *L. (V) braziliensis* and *L. (V.) guyanensis* [15]–[19]. In a preliminary study we have demonstrated that our microsatellite marker set is highly discriminatory at intra-species level. Moreover, this genotyping scheme allows the detection of mild genetic structures at different levels, and is thus, relevant for epidemiological and population genetic studies of strains within the subgenus *L.* (*Viannia*) [17].

In the present study we investigated microsatellite variation in strains of the subgenus *L.* (*Viannia*) from different Brazilian foci endemic for CL. Strains of *L. (V.) braziliensis* isolated along the Atlantic coast of Brazil and strains of *L. (V.) guyanensis* formed two clearly separated populations. Evidence for significant levels of recombination was obtained for both of these populations, and in Minas Gerais the emergence of an epidemic clone of *L. (V.) braziliensis* was identified. A third loosely associated group comprised *L. (V.) braziliensis* strains and several other subgenus *L.* (*Viannia*) species, all from northern Brazil.

## Materials and Methods

### Ethics statement

Research in this study was subject to ethical review by the European Commission and approved as part of contract negotiation for Project LeishEpiNetSA (contract 01547): the work conformed to all relevant European regulations. The research was also reviewed and approved by the ethics committee of the London School of Hygiene and Tropical Medicine (approval 5092). The *Leishmania* strains isolated from human and animal hosts and analysed in this microsatellite analysis, were received from the “Coleção de Leishmania do Instituto Oswaldo Cruz – CLIOC ([http://clioc.fiocruz.br], registered at the World Data Centre for Microorganisms under the number WDCM731 and recognized as the depository authority by the Brazilian Ministry of the Environment, MMA/CGEN Deliberação CGEN 97 de 22/03/2005, Processo 02000.003672/2004-34), and from the collection hosted by the Universidade Federal de Minas Gerais in Belo Horizonte. Only previously gathered samples from animals have been used in this study. All human strains of *Leishmania* had been isolated from patients as part of normal diagnosis and treatment with no unnecessary invasive procedures and with written and/or verbal consent recorded at the time of clinical examination. Data on human isolates were coded and anonymised.

### Parasite and DNA samples

Sources, designation, geographical origins, MLEE identification, if known, and clinical manifestation for the 120 subgenus *L.* (*Viannia*) strains from Brazil that were used in this study are listed in Table S1. These included 37 strains of *L. (V.) guyanensis*, 63 of *L. (V.) braziliensis*, four of *L. (V.) shawi*, six of *L. (V.) lainsoni*, seven of *L. (V.) naiffi*, one each of *L. (V.) utingensis* and *L. (V.) lindenbergi*, and one *L. (V.) lainsoni*/*L. (V.) naiffi* hybrid. Most of the strains were isolated from human CL cases, three from DL cases, and three strains from patients suffering from MCL. The reference strains were cloned, but all other strains represented uncloned material. Seven strains were isolated from sand fly vectors, and 18 from different animals, such as opossums (4), rodents (3), armadillos (3), dogs (2), sloths (2), pacas (2), a capuchin monkey (1) and a porcupine (1). Table 1 summarises the number of strains per species according to geographical origin, zymodeme and clinical picture.

**Table 1:** Numbers of strains per species, region, clinical picture and host of the 120 strains studied.

Species	Origin	Strains	CL	DL	MCL	Sand fly	Wild animal	Dog	nd	Zymodeme IOC/Z
*L. guyanensis* (total 37)	Amazonas	36	29			1	6			Z23
	Acre	1	1							Z110
*L. braziliensis* (total 63)	Pernambuco	14	12				2			Z45, 72–75, 78, 105
	Bahia	9	3	3	3					Z27
	Rio de Janeiro	4	4							Z27
	Espírito Santo	2						2		Z27
	Minas Gerais	15	15							nd
	Paraná	3	3							nd
	Ceará	2	1				1			Z27
	Pará	4	2			1			1	Z27
	Acre	8	8							Z78-84
	Amazonas	1	1							Z27
	Rondonia	1					1			Z53
*L. shawi* (total 4)	Pará	4				3	1			Z26
*L. lainsoni* (total 6)	Acre	3	3							Z86
	Pará	2	1				1			Z15
	Rondonia	1					1			Z15
*L. naiffi* (total 7)	Pará	5	1			1	3			Z36, 37, 38, 41
	Amazonas	2	2							Z36
*L. naiffi/L. lainsoni* hybrid (total 1)	Acre	1								Z87
*L. utingensis* (total 1)	Pará	1				1				Z101
*L. lindenbergi* (total 1)	Pará	1	1							Z102
**overall**		120	88	3	3	7	16	2	1	

VL – visceral leishmaniasis, CL – cutaneous leishmaniasis, MCL – mucocutaneous leishmaniasis, DL – disseminated cutaneous leishmaniasis, nd – not defined, IOC/Z –zymodemes according to the CLIOC system [11].

Most strains were obtained from the FIOCRUZ *Leishmania* collection (Coleção de *Leishmania* do Instituto Oswaldo Cruz, CLIOC, WDCM731, http://clioc.fiocruz.br). Seventeen strains of *L. (V.) braziliensis*, 15 from Minas Gerais and two from Pará, were obtained from the collection of the Universidade Federal de Minas Gerais, Belo Horizonte. Sample preparation and MLEE typing, based on the electrophoretic mobility of 11 enzymes in agarose gel electrophoresis, were performed as previously described [12].

DNA was isolated using proteinase K- phenol/chloroform extraction [20] or the Wizard™ Genomic DNA Purification System (Promega, Madison, WI, USA) according to the manufacturer's protocol, suspended in TE-buffer or distilled water and stored at 4°C until use.

### PCR amplification assays and electrophoretic analysis of the microsatellite markers

The standard set of 15 primer pairs (CSg46, CSg47, CSg48, CSg53, CSg55, CSg59, 6F, 7G, 10F, 11H, 11C, B3H, B6F, AC01R, and AC16R), specific for *L. (Viannia)*, was used for amplification of microsatellite containing fragments, as previously described [17]. PCRs were performed with fluorescence-conjugated forward primers. Screening of length variations of the amplified markers was done by automated fragment analysis using the ABI PRISM GeneMapper (Applied Biosystems, Foster City, CA). After manual checking the microsatellite repeat numbers were calculated for all loci by comparing the sizes of the respective fragments to those of the strains MHOM/BR/00/LTB300 (*L. braziliensis*) and MHOM/SR/87/TRUUS1 (*L. guyanensis*), which were included as reference strains in every experiment, and for which the repeat numbers had been determined by sequencing. These repeat numbers were then multiplied by two, since we have used dinucleotide microsatellites throughout, after which the size of the flanking region was added, as determined by sequencing of the reference strains. This rigorous normalization process was applied to correct for small size differences that could occur due to the use of different sequencing machines and/or fluorescent dyes during the analyses. These normalized fragment sizes for all markers were assembled into a multilocus microsatellite profile for every strain under study. In 1.7% of all loci three or four peaks were observed of which only the two most prominent bands were included in the microsatellite profiles. The microsatellite profiles of all strains analysed in this study are given in Table S1.

### Data analysis

Population structure was investigated using the STRUCTURE software [21], which applies a Bayesian model-based clustering approach. This algorithm identifies genetically distinct populations on the basis of allele frequencies. Genetic clusters are constructed from the genotypes identified, estimating for each strain the fraction of its genotype that belongs to each cluster. The following parameters were used: “burn-in” period of 20,000 iterations, probability estimates based on 200,000 Markov Chain Monte Carlo iterations. The most appropriate number of populations was determined by comparing log likelihoods for values of K between 1 and 10, with ten runs performed for each K, and by calculating ΔK, which is based on the rate of change in the log probability of data between successive K values [22].

Factorial correspondence analysis (FCA) implemented in GENETIX v 4.03 software [23] was performed, which places the individuals in a three-dimensional space according to the degree of their allelic state similarities.

Phylogenetic analysis was based on microsatellite genetic distances, calculated with the program POPULATIONS 1.2.28 (http://bioinformatics.org/~tryphon/populations) for the numbers of repeats within each locus using the Chord-distance [24], which follows the infinite allele model (IAM). Neighbor-joining trees (NJ) were constructed with the POPULATIONS software and visualized with MEGA [25]. Additionally, phylogenetic networks were inferred from the distance matrix obtained from the microsatellite dataset by using the Neighbor-Net method in SplitsTree4 [26].

Microsatellite markers as well as populations were analysed with respect to diversity of alleles (*A*), expected (gene diversity) and observed heterozygosity (*H*_e_ and *H*_o_, respectively) applying GDA (http://hydrodictyon. eeb.uconn.edu/people/plewis/software. php). *F*_IS_ is a measure of heterozygosity that assesses the level of identity within individuals compared to that between individuals. It ranges between −1 and 1, where a negative value corresponds to an excess of heterozygotes, and a positive value to heterozygote deficiency. *F*_IS_ = 0 indicates Hardy-Weinberg allele proportions. Mean *F*_IS_ estimates over loci in each population were calculated with the software FSTAT (version 2.9.3.2) [27] using Weir and Cockerman's (1984) unbiased estimators [28]. Confidence intervals per locus were assessed by randomization and bootsraping procedures over loci and individuals, implemented in GENETIX [23] using 1,000 random permutations. We also analysed the data, by computing estimates and tests of significance for various population genetic parameters. Genetic differentiation and gene flow was assessed by *F*-statistics [28]–[30] with the corresponding *P*-values (confidence test) using the MSA software [31]. Linkage between all pairs of loci in populations 1 and 2 was tested using the software ARLEQUIN, version 3.5 [32] and FSTAT [27]. *P*-values for multiple tests were corrected using a sequential Bonferroni correction to minimize the likelihood of Type 1 errors [33]. Composite digenic disequilibrium values were estimated and their significance was tested using Chi-square statistics as described by Weir [34]. An exact test for association between alleles across loci based on permutation [35] was also employed.

To assess the level of multilocus linkage disequilibrium, the Index of Association (*I_A_*, multilocus) and the *r_d_* statistic were calculated in MULTILOCUS 1.3b [36], [37]. *P* values were derived through comparison to a null distribution of 1,000 randomizations; median values were taken from 1,000 diploid resamplings of the multiallelic dataset.

## Results

### Genetic diversity of Brazilian strains of subgenus *L.* (*Viannia*)

**Figure 1:**
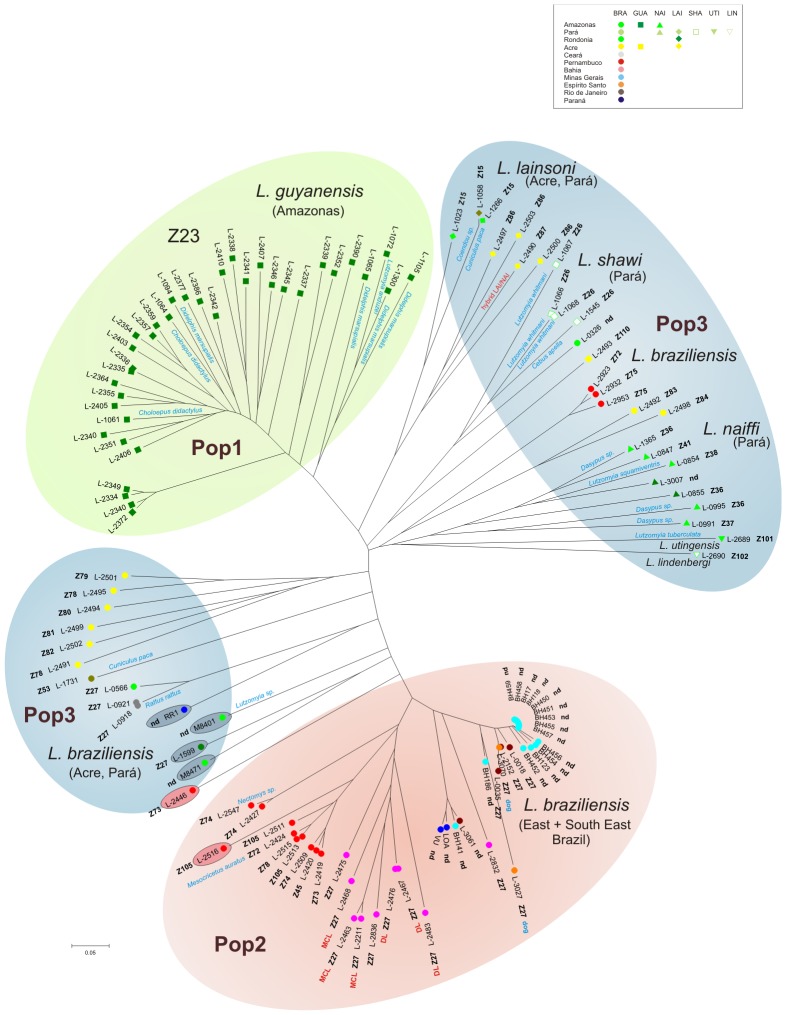
Populations and subpopulations of Brazilian strains of the subgenus of *Leishmania* (*Viannia*). A midpoint rooted Neighbour-joining (NJ) tree (radial version, rectangular version in Figure S1) was calculated for the MLMT profiles of 120 strains of different species of subgenus *L.* (*Viannia*), based on 15 microsatellite markers and using the Chord distance measure. The assignment of these strains to three main populations by the Bayesian model-based clustering approach implemented in STRUCTURE is indicated by coloured circles: population 1 (green), population 2 (red) and population 3 (blue). Strains belonging to these populations are listed in Table S1. Population 1 comprises all but one strain of *L. (V.) guyanensis* analysed in this study. Population 2 consists of 43 strains of *L. (V.) braziliensis* mainly from east Brazil. Population 3 is very diverse and includes all investigated strains of *L. (V.) lainsoni*, *L. (V.) naiffi*, *L. (V.) shawi*, *L. (V.) utingensis*, *L. (V.) lindenbergi*, 20 strains of *L. (V.) braziliensis* mainly from the north of Brazil as well as one strain of *L. (V.) guyanensis* from Acre. Putative hybrids are indicated by red or blue circles, according to their population assignment. Strain origins are indicated in the window alongside.

All 15 microsatellite markers were polymorphic in the 120 strains of the subgenus *L.* (*Viannia*) analysed here (Table 2). Taking together all strains, the number of alleles varied between 7–29, with a mean value of 15, with markers CSg47 and CSg48 being the most variable. The overall observed heterozygosity per marker ranged from 0.117 to 0.706 and was lower than the expected (0.646–0.940) for all markers. Overall inbreeding coefficients varied between 0.245 and 0.855. The discrepancy between expected and observed heterozygosity and the high *F*_IS_ values most probably reflects population substructuring (Wahlund effect) although the existence of a considerable amount of inbreeding cannot be ruled out. A total of 107 strains had unique MLMT profiles (Table S1). Five profiles, Lgua13, Lbra6, Lbra22, Lbra48 and Lsha3, were each shared by two strains. Nine of the 15 strains of *L. braziliensis* isolated between 1986 and 1992 from human CL cases in Minas Gerais presented indistinguishable MLMT profiles (Lbra26). Only two of the Minas Gerais strains were different from the predominating genotype (Figure 1, Figure S1).

**Table 2:** Characterization of the microsatellite markers for all strains and the three populations inferred by STRUCTURE.

Marker	Population *K*3	n	Repeat array	Fragment sizearray [bp]	A	*H*_e_	*H*_o_	*F*_IS_
CSg46	Pop1	36	(AC) 6–16	71–91	4	0.651	1.000	−0.548
	Pop2	43	(AC) 11–23	81–105	4	0.113	0.093	0.178
	Pop3	41	(AC) 7–22	73–103	12	0.895	0.441	0.511
	**overall**	**120**	**(AC) 6–23**	**71–105**	**14**	**0.788**	**0.486**	**0.383**
CSg47	Pop1	36	(TG) 8–18	87–107	11	0.859	0.750	0.129
	Pop2	43	(TG) 6–29	83–129	15	0.845	0.884	−0.046
	Pop3	41	(TG) 2–34	75–139	21	0.881	0.475	0.464
	**overall**	**120**	**(TG) 2–34**	**75–139**	**29**	**0.940**	**0.706**	**0.250**
CSg48	Pop1	36	(TG) 7–8	76–78	2	0.081	0.028	0.660
	Pop2	43	(TG) 4–14	70–90	4	0.524	0.070	0.868
	Pop3	41	(TG) 3–27 (34)^1^	68–117 (131)^1^	19	0.886	0.244	0.727
	**overall**	**120**	**(TG) 3–27 (34)** 1	**68–117 (131)** 1	**20**	**0.801**	**0.117**	**0.855**
CSg53	Pop1	36	(AC) 7–9	84–88	2	0.155	0.055	0.645
	Pop2	43	(AC) 7–15	84–100	3	0.402	0.256	0.367
	Pop3	41	(AC) 5–19	80–108	13	0.896	0.342	0.621
	**overall**	**120**	**(AC) 5–19**	**80–108**	**13**	**0.758**	**0.222**	**0.708**
CSg55	Pop1	36	(TG) 16–21	103–113	6	0.714	0.583	0.186
	Pop2	43	(TG) 8–13	87–97	3	0.046	0.046	−0.006
	Pop3	41	(TG) 8–20	87–111	11	0.666	0.175	0.740
	**overall**	**120**	**(TG) 8–21**	**87–113**	**13**	**0.685**	**0.252**	**0.633**
CSg59	Pop1	36	(TC) 6–8	94–98	3	0.394	0.417	−0.058
	Pop2	43	(TC) 7–8	96–98	2	0.492	0.643	−0.312
	Pop3	41	(TC) 3–10	88–102	7	0.780	0.341	0.565
	**overall**	**120**	**(TC) 3–10**	**88–102**	**7**	**0.646**	**0.471**	**0.273**
7G	Pop1	36	(AC) 5–9	88–96	3	0.133	0.139	−0.042
	Pop2	43	(AC) 5–17	88–100	4	0.389	0.349	0.104
	Pop3	41	(AC) 0–24	78–126	16	0.885	0.585	0.342
	**overall**	**120**	**(AC) 0–24**	**78–126**	**17**	**0.798**	**0.367**	**0.542**
11H	Pop1	36	(GT) 7–14	86–100	6	0.605	0.056	0.909
	Pop2	43	(GT) 7–11	86–94	3	0.298	0.116	0.613
	Pop3	41	(GT) 5–23	82–118	17	0.910	0.540	0.410
	**overall**	**120**	**(GT) 5–23**	**82–118**	**17**	**0.796**	**0.233**	**0.708**
11C	Pop1	36	(TG) 5–8	90–96	4	0.540	0.528	0.023
	Pop2	43	(TG) 3–10	86–100	4	0.525	0.395	0.249
	Pop3	41	(TG) 1–29 (42)^1^	82–138 (164)^1^	16	0.906	0.750	0.175
	**overall**	**120**	**(TG) 1–29 (42)** 1	**82–138 (164)** 1	**17**	**0.812**	**0.548**	**0.327**
6F	Pop1	36	(AC) 7–10	83–89	3	0.206	0.111	0.465
	Pop2	43	(AC) 7–12	83–93	3	0.353	0.349	0.012
	Pop3	41	(AC) 5–24	79–117	15	0.913	0.474	0.485
	**overall**	**120**	**(AC) 5–24**	**79–117**	**16**	**0.790**	**0.316**	**0.601**
10F	Pop1	36	(CA) 15–16	97–99	2	0.366	0.361	0.013
	Pop2	43	(CA) 13–21	93–109	5	0.364	0.419	−0.152
	Pop3	41	(CA) 12–21	91–109	9	0.839	0.400	0.527
	**overall**	**120**	**(CA) 12–21**	**91–109**	**9**	**0.733**	**0.395**	**0.462**
B6F	Pop1	36	(AC) 7–9	81–85	3	0.469	0.111	0.766
	Pop2	43	(AC) 6–20	79–107	11	0.690	0.738	−0.071
	Pop3	41	(AC) 2–21	71–109	14	0.855	0.300	0.652
	**overall**	**120**	**(AC) 2–21**	**71–109**	**16**	**0.855**	**0.398**	**0.535**
B3H	Pop1	36	(AC) 7–10	65–71	4	0.626	0.611	0.023
	Pop2	43	(AC) 9–18	69–87	6	0.670	0.953	−0.431
	Pop3	41	(AC) 4–22	59–95	14	0.878	0.297	0.664
	**overall**	**120**	**(AC) 4–22**	**59–95**	**14**	**0.857**	**0.638**	**0.256**
AC01R	Pop1	36	(CA) 11–17	105–117	5	0.580	0.429	0.264
	Pop2	43	(CA) 4–8	91–99	2	0.023	0.023	0.000
	Pop3	41	(CA) 2–36 (46)^1^	87–155 (175)^1^	15	0.781	0.375	0.523
	**overall**	**120**	**(CA) 2–36 (46)** 1	**87–155 (175)** 1	**16**	**0.699**	**0.263**	**0.625**
AC16R	Pop1	36	(TG) 12–15	91–97	4	0.639	0.583	0.089
	Pop2	43	(TG) 9–14	85–95	4	0.497	0.651	−0.315
	Pop3	41	(TG) 9–26	85–119	14	0.867	0.475	0.456
	**overall**	**120**	**(TG) 9–26**	**85–119**	**14**	**0.756**	**0.571**	**0.245**

N, number of strains; A, number of alleles; *H_o_*, observed heterozygosity; *H_e_*, expected heterozygosity; *F*_IS_, inbreeding coefficient;

1 Alleles which occurred in exceptional single cases are given in brackets.

More than two peaks were found for 30 of the 1800 loci (1.7%) analysed in this study. Twenty-three of the 120 strains presented such possibly aneuploid loci. Strain *L. braziliensis* L 2516 had more than two peaks in four loci, and strains *L. braziliensis* L-0018, *L. lainsoni* L-2500 and L-2503, and *L. naiffi* L-991 in two loci each. One putative aneuploid locus was seen in five strains of *L. guyanensis*, eight of *L. braziliensis*, two of *L. lainsoni*, two of *L. naiffi* and in the *L. naiffi*/*L. lainsoni* hybrid L-2490.

### Population structure of Brazilian strains of the subgenus *L.* (*Viannia*)

The multilocus microsatellite profiles consisting of the repeat numbers for 15 markers were processed using both model-based and distance-based methods and the results of both analyses are compared in Figure 1 and Figure S1.

STRUCTURE analysis assigned the 120 Brazilian strains of the subgenus *L. (Viannia)* to three main populations (Table S1, Figure S2) as inferred by Δ*K* calculation. Population 1 consisted exclusively of strains of *L. (V.) guyanensis* from the state of Amazonas (n = 36). Only one strain of *L. (V.) guyanensis*, strain L-2493 from Acre, was not part of this population. This strain was previously identified as a new enzymatic variant, Z110, of *L. (V.) guyanensis* by MLEE (unpublished results) and presented few differences in the *hsp70* sequence and RFLP [38]. Population 2 comprised 43 strains of *L. (V.) braziliensis* isolated in the eastern states of the country, namely Pernambuco, Bahia, Minas Gerais, Espírito Santo, Rio de Janeiro, and Paraná. Population 3 (n = 41) presented a mixture of 20 strains of *L. (V.) braziliensis*, the single strain of *L. (V.) guyanensis* L-2493 from Acre mentioned above, all strains of *L. (V.) shawi*, *L. (V.) lainsoni*, *L. (V.) naiffi*, the *L. (V.) lainsoni*/*L. naiffi* hybrid and the single strains of *L. (V.) utingensis* and *L. (V.) lindenbergi*. Thirty-five of these strains were isolated in the north of Brazil, five in the northeast and one in Paraná. Several strains of *L. (V.) braziliensis* had mixed membership coefficients for Populations 2 and 3, especially those from Pará (Table S1), and strain L-2446 from Pernambuco for all three populations indicating gene flow between the populations.

**Figure 2:**
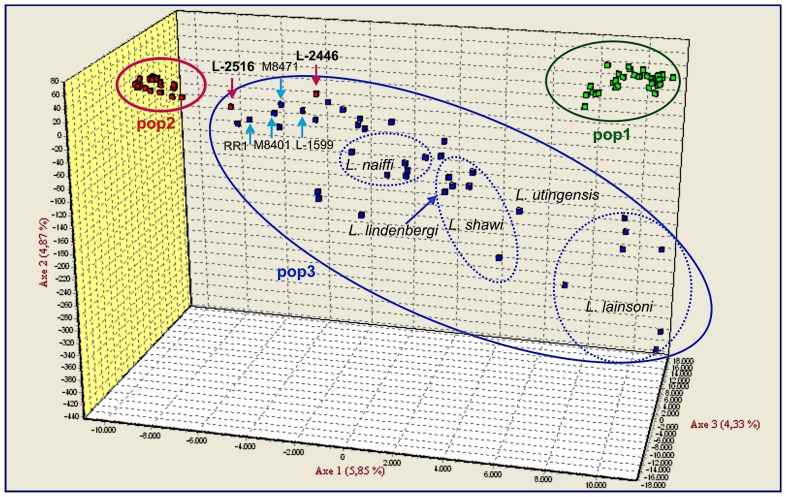
Factorial correspondence analysis (FCA) of 120 Brazilian strains of the subgenus *L. (Viannia)*. The strains labelled in green, red and blue correspond to those that were assigned, by STRUCTURE, to populations 1, 2 and 3, respectively. The two strains of Population 2, L2516 and L2446 that grouped within the blue cloud are indicated by a pink arrow. Four strains of mixed population membership, with predominating traits of population 3, are indicated by blue arrows.

**Figure 3:**
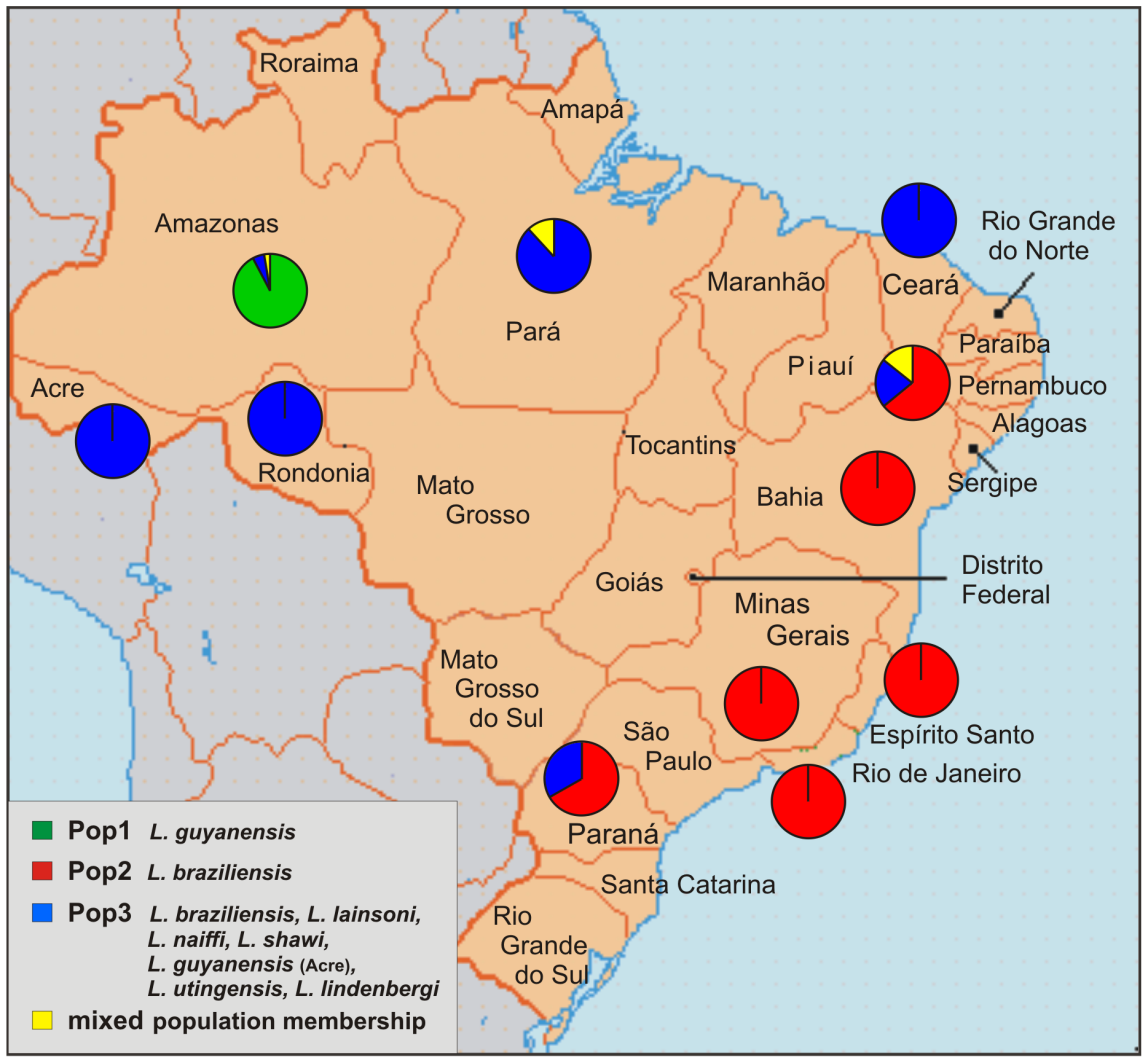
Geographical distribution of the three main populations inferred by STRUCTURE. According to the Bayesian clustering algorithm (STRUCTURE) the Brazilian strains of *L.* (*Viannia*) were assigned to three different populations, shown in green, red and blue. Pie-charts show the proportion of each population sampled in the respective geographical region. The distribution of the main populations correlates, at least partially, with the geographical origin of the strains.

The existence of Populations 1 and 2 was supported by FCA. However, strains of Population 3 were shown to be highly heterogeneous compared to Populations 1 and 2 (Figure 2). Two strains of Population 2, namely L-2516 and L-2446 both from Pernambuco, grouped within the cloud formed by Population 3. *F*-statistics revealed significant genetic differentiation between the three populations identified by STRUCTURE, especially between Populations 1 and 2 (Table 3). The correlation between population assignment and the geographical origin of the strains is shown in Figure 3.

**Table 3:** *F*_ST_ values and corresponding *p*-values for the main three populations found by STRUCTURE.

*F*_ST_-values	Pop1	Pop2	Pop3
Pop1 (36)	0	0.521	0.250
Pop2 (43)	0.0001	0	0.249
Pop3 (41)	0.0001	0.0001	0

*F*_ST_ values are in the upper triangle, *p*-values in the lower triangle. Number of strains belonging to each population is given in brackets.

The strains of *L. (V.) lainsoni* and of *L. (V.) naiffi*, including the *L. (V.) lainsoni*/*L. (V.) naiffi* hybrid, were assigned to distinct genetic groups only when Population 3 was re-analysed by STRUCTURE in order to check for hidden substructures within Population 3 (Table S1, Figure S2). Strains of *L. (V.) shawi*, however, did not present a separate entity in this analysis. When STRUCTURE was performed on Populations 1 and 2 separately, Population 1 did not show meaningful subdivision, but Population 2 was split in two sub-populations with the majority of *L. (V.) braziliensis* strains from Pernambuco and Bahia (northeast Brazil) found in 2A and strains from Minas Gerais, Espírito Santo, Rio de Janeiro and Paraná (southeast Brazil) in 2B (Table S1).

The distance analysis based on the MLMT profiles obtained for all individual strains and the inferred neighbor-joining tree (Figure 1, Figure S1) corroborated the results of FCA analysis. Populations 1 and 2 formed separate clusters corresponding to the populations observed by STRUCTURE. Strains of Population 3, however, did not appear as a monophyletic group in the tree. All strains of *L. (V.) lainsoni* were found in a small well-separated cluster. A distinct cluster was also obtained for the strains of *L. naiffi* plus the single strains of *L. (V.) utingensis* and *L. (V.) lindenbergi*, albeit with long branches. Interestingly, these strains were most closely related to five strains of *L. (V.) braziliensis*, three from Pernambuco and the two from Acre with mixed memberships for sub-populations 3A and 3D (Figure S2). The strains of *L. (V.) shawi* grouped together with the *L. (V.) braziliensis* strain L-0326 from Pará. The *L. (V.) guyanensis* strain L-2493 did not group with the other strains of *L. (V.) guyanensis*, but was closest to the strains of *L. (V.) shawi*. Another interesting cluster consisted of six relatively diverse *L. (V.) braziliensis* strains from Acre. The remaining strains of Population 3 were intermediate between the “Acre” cluster and Population 2. Most of these strains had mixed memberships in Populations 2 and 3 in the STRUCTURE analyses (Table S1) and might represent hybrids or mixed infections as cloned isolates were not used.

**Figure 4:**
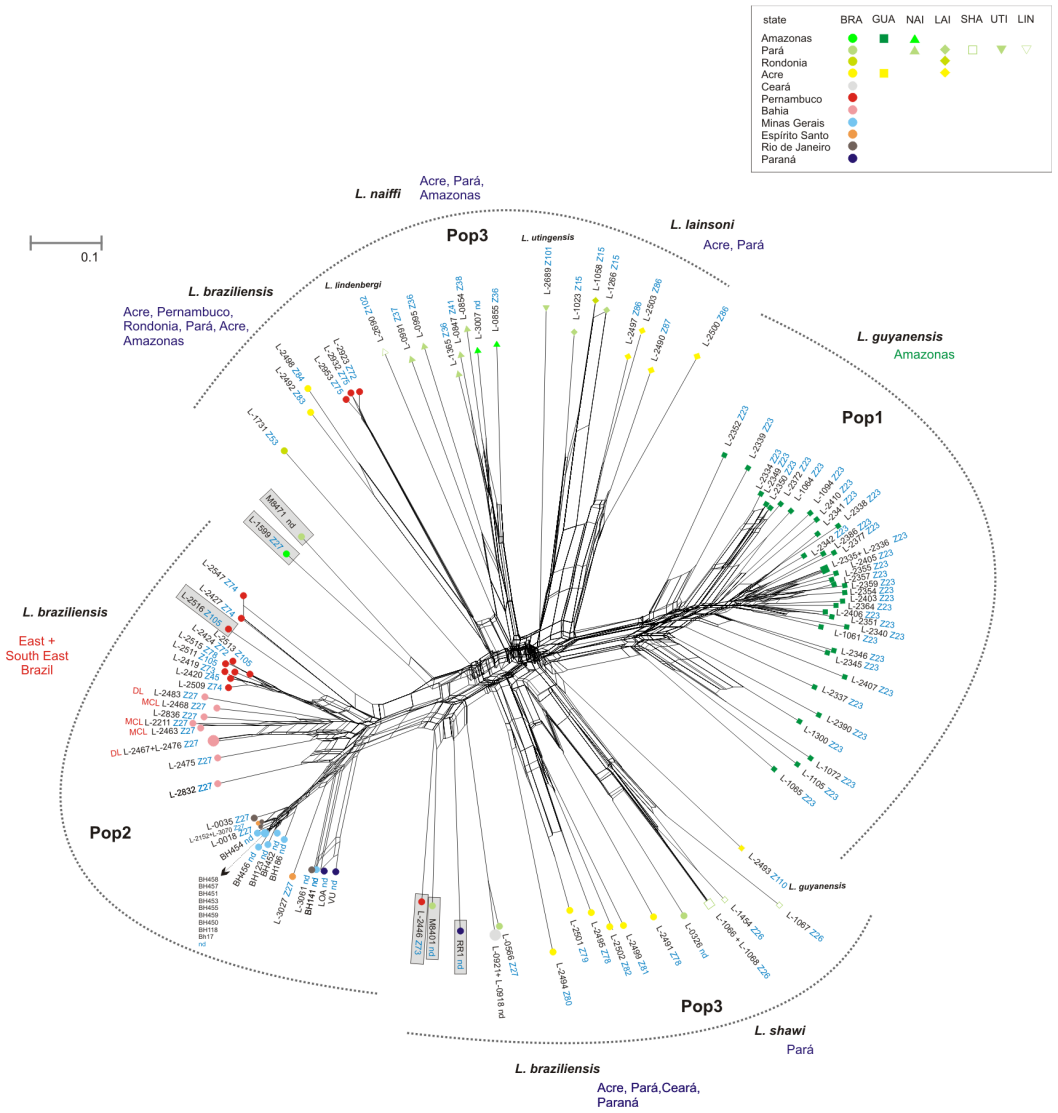
NeighborNet network based on the MLMT profiles of 120 Brazilian *L. (Viannia)* strains. The network was obtained using SplitsTree4 software and calculation of Chord distances for the 15 microsatellite markers used. The assignment of the strains to the sub-populations as inferred by STRUCTURE is indicated.

The phylogenetic NeighborNet network (Figure 4) largely confirmed the results described above. It clearly showed the tremendous diversity of the strains assigned to Population 3 and its four sub-populations. However, conflicting splits represented by boxes can be seen between and within the three main populations. The same analysis was carried out for each of the populations separately. The obtained phylogenetic networks confirm most of the sub-structures found previously by Bayesian analysis (Figures S3, S4, S5).

### Population genetics characterization of identified populations

The difference in the degree of microsatellite polymorphism between the three main populations was also reflected by mean number of alleles (MNA) which fluctuated from 4.1 to 4.9 in Populations 1 and 2, respectively, up to 14.2 in population 3 (Table 4). In Populations 1 and 2 the observed heterozygosities (Table 4) were close to those expected under Hardy-Weinberg equilibrium (HWE), whilst global *F*_IS_ values were only moderately positive for Population 1 and Population 2 (Fig. S6). Significant (*P*<0.05) excess of heterozygosity indicated by negative *F*_IS_ values was detected for only two loci, locus CSg47 in population 1 (−0.551) and locus B3H in population 2. Another 8 loci presented significant deficit of heterozygosity in a least one of the two populations. Both global *F*_IS_ values were significant (0.184 and 0.088 respectively; *P* = 0.0017 and *P* = 0.0167) and therefore panmixia must be rejected. Testing HWE within population 1 and 2 supported this scenario since both populations significantly departed from the null-hypothesis (*P*<0.002 and *P*<0.001, respectively). The Maynard Smith index of association, *I_A_*, [36], [39], which assesses multilocus linkage disequilibrium, was calculated. Another estimator, *r_d_*, was implemented because the *I_A_* tends to increase with the number of loci, a trend corrected by this statistic. To summarise, both populations displayed positive *I_A_* and *r_d_* values (1.295 and 0.097 for population 1 and 1.586 and 0.123 for populations 2, respectively) departing significantly (*P*<0.001) from panmixia (*I_A_ and r_d_* = 0).

**Table 4:** Population genetics indices of the three main populations detected by STRUCTURE.

Pop	Species	Region	N	P	MNA	*H*_e_	*H*_o_	*F*_IS_^a^	*F_IS_^b^*
Pop1	*L. guyanensis* (36/37)	Amazonas (36/36)	36	1	4.1	0.468	0.384	0.181	0.184
Pop2	*L. braziliensis* (43/63)	**Bahia (9/9),**	43	1	4.9	0.415	0.399	0.040	0.088
		**Pernambuco (11/14),**							
		**Rio de Janeiro (4/4)**							
		**Espírito Santo (2/2)**							
		**Minas Gerais (15/15),**							
		**Paraná (2/3)**							
Pop3	*L. guyanensis* (1/37),	**Acre (1/1)**	41	1	14.2	0.856	0.414	0.519	n.d.
	*L. braziliensis* (20/63),	**Rondonia (1/1),**							
		Amazonas (1/1),							
		**Ceará (2/2),**							
		Pernambuco (3/14),							
		Paraná (1/3)							
		**Pará (4/4)**							
		**Acre (8/8)**							
	*L. shawi* (4/4),	**Pará**							
	*L. naiffi* (7/7),	**Pará, Amazonas**							
	*L. lainsoni* (6/6),	**Acre, Pará, Rondonia**							
	*L.nai./L.lain.*hybr. (1/1)	**Acre**							
	*L. utingensis* (1/1),	**Pará**							
	*L. lindenbergi* (1/1)	**Pará**							
**Overall**			120	1	15.5	0.781	0.399	0.490	n.d.

N, number of strains; P, proportion of polymorphic loci; MNA, mean number of alleles; *H_o_*, observed heterozygosity; *H_e_*, expected heterozygosity; *F_is_*, inbreeding coefficient, *F_is_* ^a^ all strains of the data set were included in the calculations, *F_is_* ^b^ only one strain per genotype was included in the calculations; n.d., not done.

Predominating regions are marked by bold letters, normal letters are used for regions for which single strains are found in the respective population.

However, the *F*_IS_ values gathered from populations 1 and 2 are consistently lower than those observed in previous reports [40], [41], indicating that gene conversion or recombination may play a substantial role in *Viannia* species present in the Amazon Basin and along the Atlantic coast and to a lesser extent westward from the Andes. In contrast, in Population 3 the observed heterozygosity was much less than the expected resulting in a high *F*_IS_ value, most probably due to population subdivision (Wahlund effect) although high rates of gene conversion or inbreeding cannot be excluded. This group was not tested for all population genetic parameters, since it represents a composite and artificial unit.

In order to test whether associations between the 15 microsatellite loci are in gametic equilibrium in populations 1 and 2, as expected for random-mating populations, we have applied both the composite disequilibrium test and the exact test to all 105 pairwise comparisons. For population 1, only one strain representing the microsatellite profile that was shared by two strains (Table S1) was included in the data matrix. Using Chi-square test and exact test, 24 and 37 significant associations were respectively detected. However, after Bonferroni correction, those numbers dropped to 6 and 10. In population 2 nine strains of *L. braziliensis* from Minas Gerais shared an identical genotype, Lbra26, and further four strains had highly related genotypes, Lbra 28, 29, 30 and 31. These strains most probably represent an epidemic clone and only one Lbra 26 strain was therefore included in the data matrix for linkage disequilibrium calculations. The Chi-square test and the exact test revealed 30 and 49 significant associations (28 and 45 after Bonferroni correction), respectively for population 2. The three populations detected according to the Bayesian algorithm were also significantly separate from each other with highly significant *F_st_* values ranging from 0.249 to 0.521 (Table 3).

## Discussion

Because of its high resolution potential, its reproducibility and the possibility of data storage, multilocus microsatellite typing (MLMT) is currently the most widely used approach for strain level differentiation in the genus *Leishmania*. Analysis of length polymorphisms of microsatellite-containing sequences has recently revealed geographical and hierarchical population structures in different *Leishmania* species such as *L. (L.) tropica*, *L. (L.) major* and the *L. (L.) donovani* complex (for review see [14]), the predominance of inbreeding in *L. (V.) braziliensis* and *L. (V.) guyanensis* [40], [41], and confirmed that the agent of VL in the NW is *L. (L.) infantum*, which has been recently imported multiple times from southwest Europe to the New World [42]. In the present study, a MLMT approach employing 15 microsatellite markers previously shown to be highly discriminatory for strains of the subgenus *L. (Viannia)* [17], was used to explore the genetic diversity of 120 strains from Brazil in order to unravel discrete populations.

### Population structure of Brazilian strains of the subgenus *L.* (*Viannia*)

Different types of population genetic analyses, including Bayesian inference (as implemented in STRUCTURE), distance-based (NJ and Neighbor Net in SplitsTree) and factorial correspondence analysis as well as *F* statistics revealed the existence of two well-defined populations in the sample set, namely Population 1 consisting of all but one strain of *L. (V.) guyanensis* from the Amazonas state and Population 2 comprising 43 strains of *L. (V.) braziliensis* from Eastern Brazil.

STRUCTURE identified a third population including 20 strains of *L. (V.) braziliensis*, one of *L. (V.) guyanensis* and the strains belonging to other species, most of which were isolated in the north of the country. When the strains of *L. (V.) lainsoni*, *L. (V.) naiffi, L. (V.) shawi*, *L. (V.) utingensis* and *L. (V.) lindenbergi* were excluded from the data set, the strains of *L. (V.) braziliensis* were assigned to the same Populations 2 and 3 (data not shown). The Population 3 identified in the complete data set was, however, not well supported by the distance and FCA analyses which showed that the strains of this population are highly diverse and only distantly related to each other as can be clearly seen in the NeighborNet network produced in SplitsTree (Figure 4). When re-analysed by STRUCTURE, Population 3 split into four sub-populations (Figure S2) and strains of *L. (V.) lainsoni* (3C) and *L. (V.) naiffi* (3D) were assigned to separate sub-populations. Two strains of *L. (V.) braziliensis* from Acre (L-2492 and L-2498) were found to be putative *L. (V.) braziliensis/L. (V.) naiffi* hybrids, as previously suggested [38], and were also assigned to the “*L. (V.) naiffi*” sub-population. However, strains of *L. (V.) shawi* grouped together with ten strains of *L. (V.) braziliensis*, mainly from Acre, strain L-2493 of *L. (V.) guyanensis* and the strains of *L. (V.) utingensis* and *L. (V.) lindenbergi* (3A), and the fourth sub-population (3B) consisted of *L. (V.) braziliensis* from Pernambuco, Ceará, Pará, Paraná and Amazonas. This last group seems, however to be rather artificial, since seven of the ten strains have hybrid genotypes sharing alleles that are specific for Population 2 and 3, respectively. Strains of *L. (V.) braziliensis* of population 3 are considerably different from each other and very distinct from those that were assigned to Population 2, except those having mixed memberships to Populations 2 and 3. Whether such strains represent outliers, as stated in a different study that used AFLP for typing strains of *L. (V.) braziliensis* and *L. (V.) peruviana* mainly from Peru and Bolivia [43], or different taxa requires further investigations including additional strains and using DNA sequence-based comparisons.

NeighborNet analyses provide a snapshot overview of the general structure in the data and are useful as a guide for further analysis [44]. The phylogenetic network obtained here for the full sample set is distinctly non-treelike and demonstrates marked ambiguity in the signal. Only Populations 1 and 2 formed distinct clusters in the network. The reticulate patterns seen in the network between (Figure 4), and within the three main populations (Figures S3, S4, S5) could result from hybridization, recombination events or gene conversion.

For the strains assigned by STRUCTURE to Population 3 no clear structuring is seen in the NeighborNet network obtained for the whole data set (Figure 4), maybe with the exception of the strains of *L. (V.) lainsoni* which seem to represent a distinct lineage in the network. When the NeighborNet analysis was performed for Population 3 only, strains of *L. (V.) lainsoni*, *L. (V.) shawi* and *L. (V.) naiffi* were assigned to distinct clusters although conflicting signals were still detectable (Figure S5). The independent status of *L. shawi* and its apparent affiliation with *L. (V.) braziliensis* by this high resolution microsatellite analysis does not agree with results of MLST analysis, in which *L. (V.) shawi* and *L. (V.) guyanensis* are not resolved as separate entities [45]. MLEE, PCR-RFLP of the ribosomal ITS and sequencing of the *hsp*70 had already previously suggested that *L. (V.) guyanensis and L. (V.) shawi* were closely related [12], [13], [38], strains of *L. (V.) braziliensis* from northeastern Brazil belonging to zymodeme Z75 were however, found to be related to *L. (V.) shawi* [46] which is in agreement with the results of our MLMT study. Assessment of the taxonomic status of *L. shawi* thus warrants further investigation with more extensive DNA sequence comparisons. All strains of population 3 and only those are found on long branches in the overall network. Because NeighborNet is prone to long-branch attraction, rapidly evolving lineages can be inferred as being closely related regardless of their evolutionary relationships. Whether these strains have a high mutation rate leading to numerous homoplasies or to convergence, which could be misinterpreted as having evolved once in a common ancestor, remains to be established.

Given its high genetic diversity, Population 3 could represent the ancestral lineage and might have given rise to two new populations through bottleneck events (Populations 1 and 2). The Amazon forest seems to be the central distribution area with secondary spreads to the northeast, east and south. This would be consistent with the hypothesis of an Amazon origin of CL in Brazil, with later spread to other regions, most probably through human migrations [6]. However, we cannot exclude that sampling biases are responsible for the weak resolution of the strains in Population 3. More extensive sampling in the north of Brazil, where most of these strains were isolated, is needed to address these questions.

### Genetic diversity of Brazilian strains of the subgenus *L.* (*Viannia*)

Almost all strains investigated in this study presented unique microsatellite profiles, except 13 strains from Minas Gerais that had identical or highly similar microsatellite profiles and might have been isolated during an outbreak of CL in this area.

Previous studies using isoenzyme typing [12] or PCR-RFLP of the ribosomal internal transcribed spacer [13] have already demonstrated that *L. (V.) braziliensis* is much more polymorphic than *L. (V.) guyanensis*. Furthermore, strains of *L. (V.) braziliensis* from the Amazon region and from Pernambuco were shown to present the highest level of genetic diversity whereas those from Rio de Janeiro were more homogeneous [11[,[46[. In our study the MNA (Table 4) was similar for strains of *L. (V.) guyanensis* (Population 1) and of *L. (V.) braziliensis* from east Brazil (Population 2) indicating a similar level of genetic diversity for these groups of strains. For *L. (V.) braziliensis* strains of Population 3, MNA and thus genetic heterogeneity was however, considerably higher. This confirms that the previously described greater diversity of *L. (V.) braziliensis* is related to the genetically heterogeneous strains from the north of Brazil.

The strains of *L. (V.) guyanensis* investigated herein were more diverse than expected considering that they all belonged to the same zymodeme Z23 which is in agreement the results of a recent MLST study [45]. Only two strains, MHOM/BR/1997/203P and MHOM/BR/1997/203G, isolated from the skin (P) and a lymph node (G) of the same patient, shared an identical MLMT profile (), all other strains presented different patterns of microsatellite variation. Previous studies using monoclonal antibodies had already pointed to the existence of two distinct sub-populations in the Brazilian Amazon region [47]. In our study, the existence of two *L. (V.) guyanensis* sub-populations in the Amazonas state (data not shown) was not supported by the genetic distance analyses. Our MLMT approach confirmed however, the existence of a new *L. (V.) guyanensis* genotype in Acre, where this species is not commonly found. This strain was very closely related to *L. (V.) shawi* as previously suggested [38]. In French Guyana, two distinct populations of *L. (V.) guyanensis* had been studied using a PCR-RFLP approach targeting ribosomal DNA sequences and found to have originated from two ecologically different regions and to differ in clinical manifestations of CL [48]. In a previous study comparing microsatellite variation in a limited number of strains of *L. (V.) guyanensis* from Brazil, Peru, Suriname and French Guyana, the strains grouped according to their geographical origin [17]. Future investigations should include strains sampled in different locations, since it has been speculated recently that strains from the eastern and southern Brazilian Amazon region might represent different genetic groups of *L. (V.) guyanensis* [*8*].

We found only weak correlations between the MLMT profiles and the results of previous isoenzyme typing (Table S1, Table S2). As already mentioned above, the strains of *L. (V.) guyanensis*, despite all being of zymodeme Z23, could be individualised by MLMT but were all grouped in Population 1, with the exception of the single strain from Acre. Strains of *L. (V.) braziliensis* with identical isoenzyme patterns were assigned to different populations or genetic groups by MLMT. The majority of the strains presenting the predominant zymodeme Z27 grouped in Population 2 but those with hybrid genotypes were found in Population 3B. This implies that zymodeme Z27 is paraphyletic and does not reflect the genetic diversity of the strains which was also shown by the MLST study [45].

We did not find any correlation between a particular MLMT profile and the clinical presentation of the disease. The two strains isolated from MCL patients in Bahia grouped together with strains from CL cases from the same area, although the long term outcome of those CL cases is not known. This is consistent with previous studies which suggested that the clinical outcome of the disease caused by *L. (Viannia)* parasites is also influenced by host genetic and/or immune factors [49]–[51] which could possibly be stimulated through pre-exposure to sand fly saliva [52]. In conclusion, the only correlation found for MLMT patterns of the *L. (Viannia)* strains studied herein was that to their geographical origin. Similar observations were published earlier for *L. (V.) braziliensis* [11] and *L. (L.) infantum* in Brazil [53] and might be associated with different transmission cycles with different sand fly vectors and/or animal reservoirs involved in those areas.

### Reproductive strategies among Brazilian strains of the subgenus *L.* (*Viannia*)

Despite the fact that recombination in *Leishmania* has been proved to occur *in vitro* in the sand fly hosts [54], [55] and the growing evidence of gene flow coming from different population genetic studies using MLST and MLMT approaches (reviewed in [14]), *Leishmania* species are still considered as predominantly clonal organisms [56]. Especially in the case of strains of the subgenus *L.* (*Viannia*) this hypothesis has been challenged by the frequent detection of hybrids involving different species of the *L. (Viannia)* subgenus indicating that recombination events are much more frequent in these parasites than previously thought [12], [57]. *L. (V.) braziliensis/L. (V.) peruviana* hybrids have been identified by microsatellite typing and found to be quite common in Peruvian foci where both species can occur sympatrically [58] and *L. (V.) braziliensis/L. (V.) guyanensis* hybrids are not uncommon [59]–[61]. Hybrid MLEE profiles have been observed for *L. (V.) laisoni/L. (V.) naiffi, L. (V.) braziliensis/L. (V.) naiffi, L. (V.) braziliensis/L. (V.) guyanensis* in Brazil [62], and these hybrids had been mostly isolated from patients living in areas with sympatric circulation of both putative parental species. In our study, some strains had mixed membership of the different populations identified and were considered to be putative hybrids, although this will have to be confirmed by analysis of cloned parasites. A recently published MLSA study of subgenus *L.* (*Viannia*) strains has also provided evidence for recombination occurring in both *L. braziliensis* and *L. guyanensis* [45].

Strong linkage disequilibrium, or non-random association of genotypes at different loci, and a distinct phylogenetic signal are the criteria for the identification of clonality [56]. To start with the latter, strong phylogenetic signals are clearly absent in both the NJ distance tree (Figure 1 and Figure S1) and the NeighborNet network (Figure 4). Calculation of linkage disequilibrium which does not depend on the ploidy status [56] revealed a higher number of significant associations between loci for both populations 1 and 2 than what would be expected due to chance for a random mating population. On the other hand, the majority of the pairwise comparisons were not significant and many more loci appear to be recombining than would be expected for a strictly clonal population. We can, however not exclude that population subdivision (Wahlund effect) accounts, at least partially, for the amount of disequilibrium found for populations 1 and 2. Taking in consideration the limited linkage disequilibrium, the absence of overrepresented genotypes and the weak phylogenetic signal observed in this study, we would conclude that recombination has an important impact in populations 1 and 2. The MLMT approach used here, however also identified an epidemic clone consisting of 13 strains of *L. braziliensis* isolated between 1986 and 1992 from human CL cases in Minas Gerais. Nine of these strains shared an identical microsatellite profile (Lgua26) and the profiles of the other four strains differed from profile Lgua26 in only one locus each.

Different levels of clonality versus recombination have been earlier suggested to occur within some bacterial and protozoan species due to variation in geographic sampling [36] and this might be also the case for species of the subgenus *L.* (*Viannia*). The substantial heterozygote deficiency and extreme inbreeding found by MLMT analysis of *L. (V.) braziliensis* from Bolivia and Peru was consistent with a predominantly endogamic mode of reproduction (mating with relatives) with occasional recombination events between individuals of different genotypes [40]. In contrast, significant homozygosity and only little linkage disequilibrium was observed for populations of *L. (V.) guyanensis* from French Guyana suggesting a high level of sexual recombination and substantial endogamy [41]. Ramirez et al. [63] stated that heterozygosity statistics at microsatellite loci has to be interpreted with caution in the context of parasite sexuality because strong linkage disequilibrium can be accompanied by negative and positive *Fis* values. In our study, mild linkage disequilibrium was observed together with relatively low *Fis* values, compared to those previously published [40], [41]. Indeed the *Fis* values observed for the *L. guyanensis* strains from the Brazilian Amazon region (0.184) and for the *L. braziliensis* strains from Eastern Brazil (0.088) are below the values observed in former publications (0.278 and 0.307) and this is without partitioning steps to correct for a potential Wahlund effect, which might deflate this index. Consequently, the selfing rates for the considered populations are likely to be ≪0.50. This implies that clonality, selfing and random union of gametes contribute to the shaping of *Viannia*'s natural populations. However, one of the future challenges is to understand why the contribution of sex is more significant in the Amazon Basin.

The analyses used for calculations of heterozygosity are based on the assumption of diploidy. Recently, whole genome sequencing and FISH analyses have, however confirmed significant chromosomal copy number variations for different species of *Leishmania* [64]–[68]. In the only *L. braziliensis* strain, MHOM/BR/75//M2904, that has been fully sequenced so far, 30 of the 35 chromosomes were clearly triploid, three were tetrasomic and one hexasomic [66]. Whether other strains of *L. braziliensis* show similar or different ploidy patterns, as shown for all other *Leishmania* species examined so far [64], [66], , remains to be established. More than two alleles have been observed for only 1.7% of the microsatellite loci analysed in this study. This could possibly be due to aneuploidy [64], [66], although other reasons such as mixed strains, duplication or stutter bands cannot be excluded. Sterkers at al. ([64], [65] were able to show that in *L. major* chromosomal content varies not only from strain to strain but also from cell to cell creating ‘mosaic aneuploidy’. This leads to high karyotypic diversity and conserved intra-strain genetic heterogeneity combined with loss of heterozygosity per cell. The total number of alleles can, however be maintained in a strain. As a consequence, DNA-based typing methods, including the microsatellite typing approach used herein, cannot decide if a cell population (or strain) consists of heterozygous cells or of homozygous cells presenting different allelic and ploidy content [65].

In conclusion, this study showed the extensive microsatellite diversity present in the subgenus *L. (Viannia)* and indicated that *L. (V.) braziliensis* and, to a lesser extent, *L. (V.) guyanensis* exhibit features indicative of both clonality and recombination. Recombination could explain the tremendous genetic diversity and limited population structure. The genetic heterogeneity of Brazilian strains of different species of the subgenus *L. (Viannia)* was found to be higher than previously shown by techniques such as isoenzyme typing and PCR-RFLP approaches. The different clustering approaches used in this study identified two different genetic groups or populations, one consisting of *L. (V.) guyanensis* strains from the Amazon region and the other of *L. (V.) braziliensis* strains from the southeast of Brazil, clearly differentiated from the other investigated strains. *L. (V.) braziliensis* strains from the north of Brazil did not group with those from the Atlantic coast but were found to be very polymorphic. These strains seemed to be more closely related to the strains of *L. (V.) shawi*, *L. (V.) naiffi*, and *L. (V.) lainsoni* also isolated in northern Brazilian CL foci. All findings concerning the strains from northern Brazil may, however, be subject to bias due to an inadequate sampling strategy. More strains need to be sampled from this region in order to fine tune the population structure of these parasites and their mode of reproduction.

## Supporting Information

Figure S1 Rectangular NJ tree showing the populations and subpopulations of 120 Brazilian *L.* (*Viannia*) strains. A midpoint rooted Neighbour-joining (NJ) tree (rectangular version) was calculated for the MLMT profiles of 120 strains of different species of the subgenus *L.* (*Viannia*), based on 15 microsatellite markers and using the Chord distance measure. The assignment of these strains to three main populations by the Bayesian model-based clustering approach implemented in STRUCTURE is indicated by colored branches: population 1 (green), population 2 (red) and population 3 (blue). Strains belonging to these populations are listed in Table S1. Population 1 comprises all but one strain of *L. (V.) guyanensis* analysed in this study. Population 2 consists of 43 strains of *L. (V.) braziliensis* mainly from east Brazil. Population 3 is very diverse and includes all investigated strains of *L. (V.) lainsoni*, *L. (V.) naiffi*, *L. (V.) shawi*, *L. (V.) utingensis*, *L. (V.) lindenbergi*, 20 strains of *L. (V.) braziliensis* mainly from the north of Brazil as well as one strain of *L. (V.) guyanensis* from Acre. Putative hybrids are indicated by red or blue circles, according to their population assignment. Strain origins are indicated in the window alongside.
(TIF)

Figure S2 Population structure of the 120 strains inferred by Bayesian analysis with STRUCTURE. The Bayesian algorithm assigned the 120 Brazilian strains of subgenus *L. (Viannia)* to three populations. Population 1 (green) comprises all but one strain of *L. (V.) guyanensis* analysed in this study. Population 2 (red) consists of 43 strains of *L. (V.) braziliensis* mainly from eastern Brazil. Population 3 (blue) is very diverse and includes all investigated strains of *L. (V.) lainsoni*, *L. (V.) naiffi*, *L. (V.) shawi*, *L. (V.) utingensis*, *L. (V.) lindenbergi*, 20 strains of *L. (V.) braziliensis* mainly from the north of Brazil as well as one strain of *L. (V.) guyanensis* from Acre. Four sub-populations are distinguished in Population 3 when STRUCTURE was re-run separately for the strains of this population. Sub-population 3A comprises all strains of *L. (V.) shawi*, eight strains of *L. (V.) braziliensis* (6 from Acre, one from Pará and one from Rondonia), the single strain of *L. (V.) guyanensis* from Acre, *L. (V.) utingensis* and *L. (V.) lindenbergi*; 3B ten strains of *L. (V.) braziliensis* (3 from Pernambuco, 3 from Pará, 2 from Ceará and single strains from Paraná and Amazonas); 3C all strains of *L. (V.) lainsoni*; and 3D all strains of *L. (V.) naiffi* and two putative *L. (V.) braziliensis/L. (V.) naiffi* hybrids.
(TIF)

Figure S3 NeighborNet network of Population 1 as inferred by STRUCTURE. Six strains were isolated from animal hosts, two from *Choloepus didactylus* and four from *Didelphis marsupialis*, and one from a sand fly, *Lutzomyia anduzei*, all other strains were isolated from human CL cases.
(TIF)

Figure S4 NeighborNet network of Population 2 as inferred by STRUCTURE. Four strains were isolated from animal hosts, two from dogs and one each from *Nectomys sp.* and *Mesocricetus auratus*, three from human MCL and three from human DL cases, all other strains were isolated from human CL cases. The assignment of the strains to the two sub-populations of Population 2, A and B, is indicated. Strains presenting mixed membership coefficients in two sub-populations are highlighted in grey. The two sub-populations are largely confirmed by the phylogenetic network albeit some strains occur at intermediate positions.
(TIF)

Figure S5 NeighborNet network of Population 3 as inferred by STRUCTURE. Eight strains were isolated from animal hosts, three from *Dasypus sp.*, two from *Cuniculus paca* and one each from *Coendou sp.*, *Cebus apella* and *Rattus rattus*, five from sand flies, three from *Lutzomyia whitmani* and one each from *L. tuberculata, L. squamiventris* and *Lutzomyia sp.*, all other strains were isolated from human CL cases. The four sub-populations of Population 3, A, B, C and D, are indicated. The phylogenetic network confirms the assignment of strains of *L. (V.) lainsoni* to sub-population C and that of strains of *L. (V.) naiffi* to sub-population D. The sub-populations A and B are not well supported in this NeighborNet network. Strains of *L.* (V.) *shawi* were found on a separate branch together with one strain of *L. (V.) braziliensis* from Pará, L-0326, and the single strain of *L. (V.) guyanensis* from Acre, L-2493.
(TIF)

Figure S6 Calculation of inbreeding coefficients for Populations 1 and 2. *F_is_* values were calculated for each of the 15 loci and over all loci. Strains with identical genotypes were excluded from the analyses to avoid medical driven sampling bias and clones over-representation. For each locus, 95% confidence intervals (CI) were obtained by bootstrapping over loci (GENETIX). *P* values are indicated by stars (*P*<0.05).
(PDF)

Table S1 Designation, characteristics and MLMT profiles of the Brazilian strains of subgenus *Leishmania (Viannia)* used in this study. ^T^ – reference strain of the species; ^1^ – zymodemes according to the CLIOC system – IOC/Z [11]; ^2^ – population assignment according to STRUCTURE analysis; ^3^ – normalization of microsatellite fragment sizes in described as Materials and Methods. VL – visceral leishmaniasis; CL – cutaneous leishmaniasis; MCL – mucocutaneous leishmaniasis; DL – disseminated cutaneous leishmaniasis; CLIOC - Coleção de Leishmania do Instituto Oswaldo Cruz (IOC-L-code); BH - Coleção de Universidade Federal de Minas Gerais, Belo Horizonte, Brazil.
(XLSX)

Table S2 Distribution of zymodemes and animal hosts for the populations and sub-populations found by MLMT.
(DOCX)
