# Supplementary figures and images for: Population Structure and Evidence for Both Clonality and Recombination among Brazilian Strains of the Subgenus Leishmania (Viannia)
Source: PLoS Negl Trop Dis. 2013 Oct 31;7(10):e2490. doi: 10.1371/journal.pntd.0002490 (PMC3814519; doi:10.1371/journal.pntd.0002490)

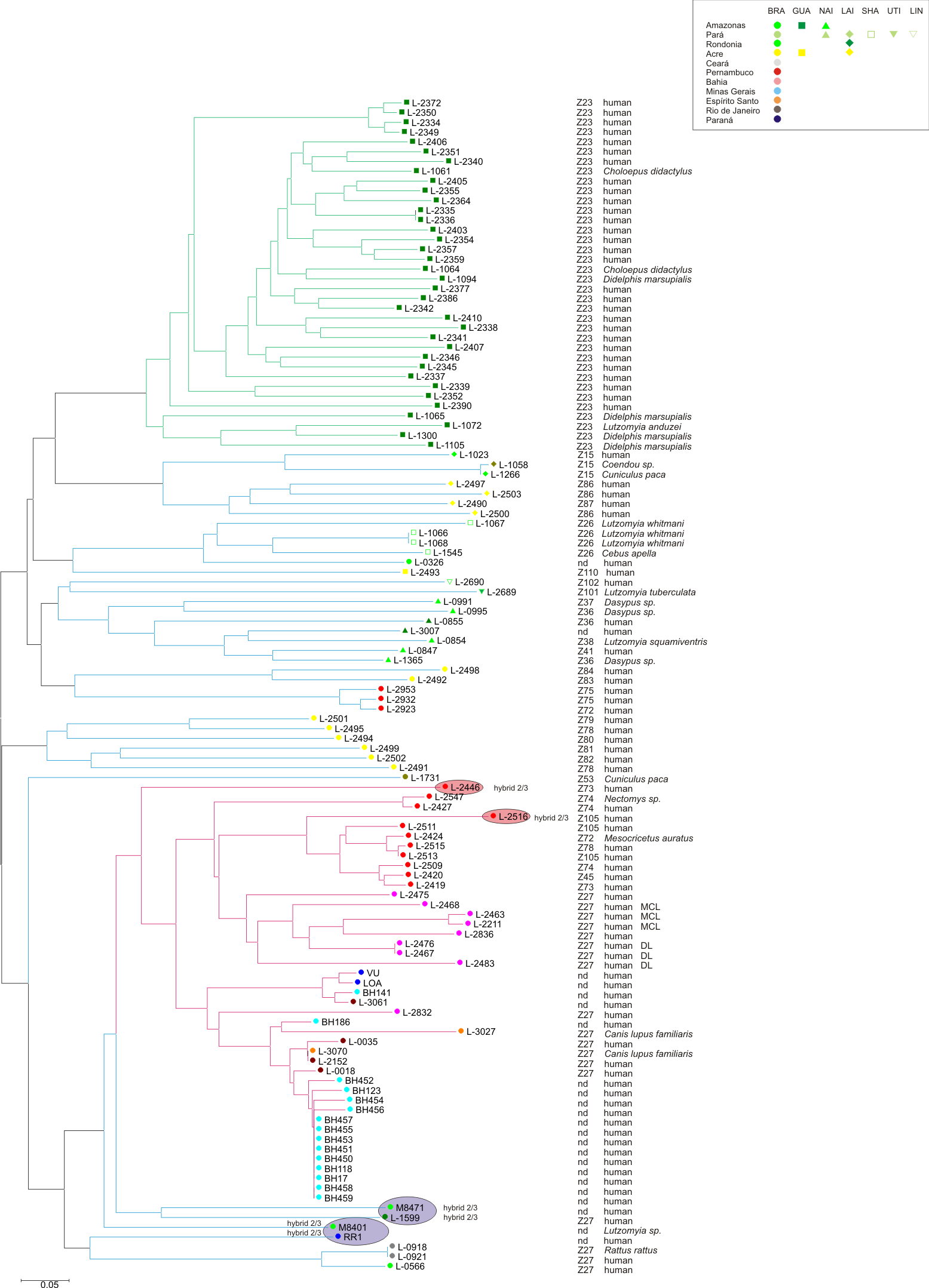

Supplement: Figure S1 — Rectangular NJ tree showing the populations and subpopulations of 120 Brazilian L. (Viannia) strains. A midpoint rooted Neighbour-joining (NJ) tree (rectangular version) was calculated for the MLMT profiles of 120 strains of different species of the subgenus L. (Viannia), based on 15 microsatellite markers and using the Chord distance measure. The assignment of these strains to three main populations by the Bayesian model-based clustering approach implemented in STRUCTURE is indicated by colored branches: population 1 (green), population 2 (red) and population 3 (blue). Strains belonging to these populations are listed in Table S1. Population 1 comprises all but one strain of L. (V.) guyanensis analysed in this study. Population 2 consists of 43 strains of L. (V.) braziliensis mainly from east Brazil. Population 3 is very diverse and includes all investigated strains of L. (V.) lainsoni, L. (V.) naiffi, L. (V.) shawi, L. (V.) utingensis, L. (V.) lindenbergi, 20 strains of L. (V.) braziliensis mainly from the north of Brazil as well as one strain of L. (V.) guyanensis from Acre. Putative hybrids are indicated by red or blue circles, according to their population assignment. Strain origins are indicated in the window alongside. (TIF) [file pntd.0002490.s001.tif]

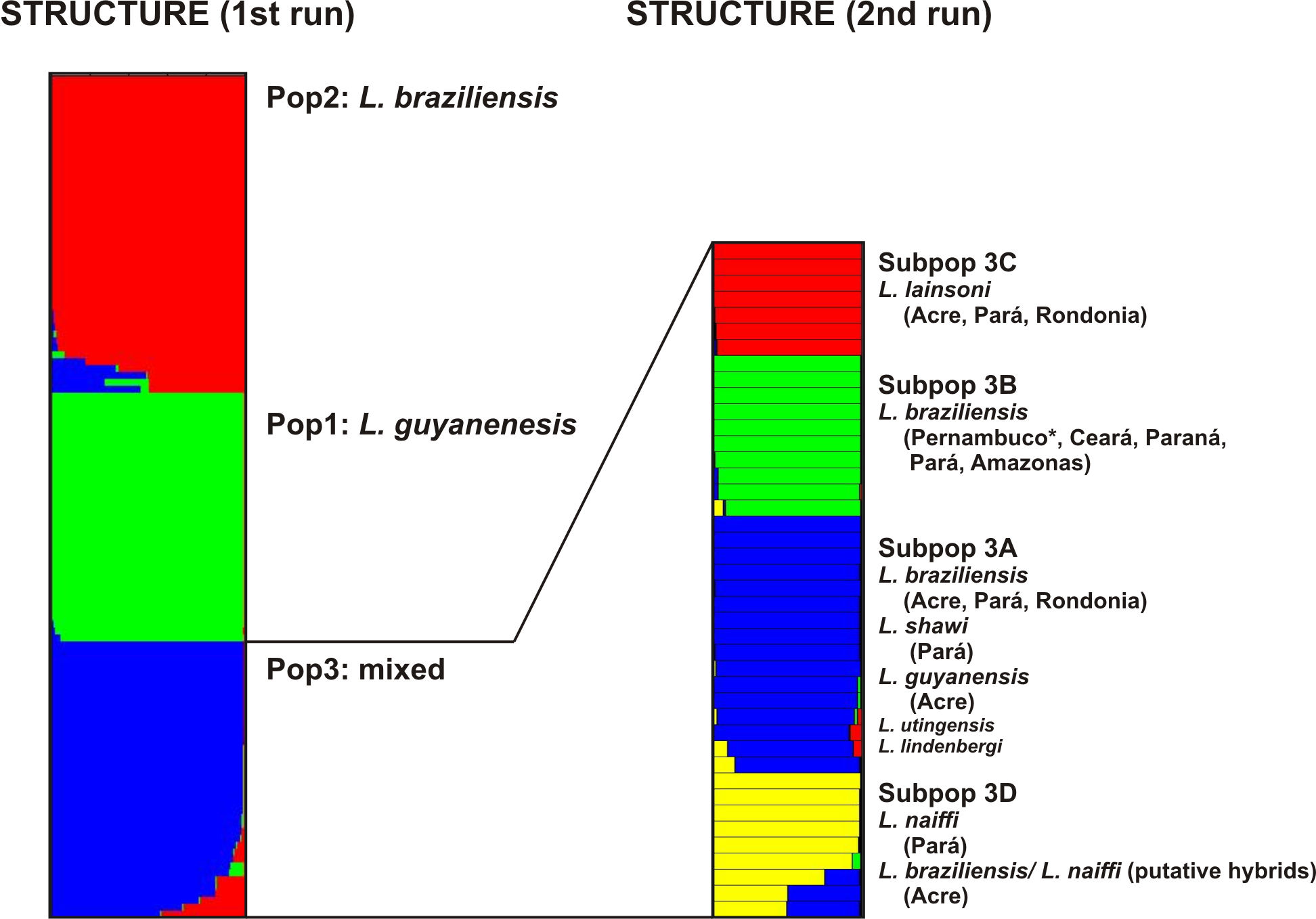

Supplement: Figure S2 — Population structure of the 120 strains inferred by Bayesian analysis with STRUCTURE. The Bayesian algorithm assigned the 120 Brazilian strains of subgenus L. (Viannia) to three populations. Population 1 (green) comprises all but one strain of L. (V.) guyanensis analysed in this study. Population 2 (red) consists of 43 strains of L. (V.) braziliensis mainly from eastern Brazil. Population 3 (blue) is very diverse and includes all investigated strains of L. (V.) lainsoni, L. (V.) naiffi, L. (V.) shawi, L. (V.) utingensis, L. (V.) lindenbergi, 20 strains of L. (V.) braziliensis mainly from the north of Brazil as well as one strain of L. (V.) guyanensis from Acre. Four sub-populations are distinguished in Population 3 when STRUCTURE was re-run separately for the strains of this population. Sub-population 3A comprises all strains of L. (V.) shawi, eight strains of L. (V.) braziliensis (6 from Acre, one from Pará and one from Rondonia), the single strain of L. (V.) guyanensis from Acre, L. (V.) utingensis and L. (V.) lindenbergi; 3B ten strains of L. (V.) braziliensis (3 from Pernambuco, 3 from Pará, 2 from Ceará and single strains from Paraná and Amazonas); 3C all strains of L. (V.) lainsoni; and 3D all strains of L. (V.) naiffi and two putative L. (V.) braziliensis/L. (V.) naiffi hybrids. (TIF) [file pntd.0002490.s002.tif]

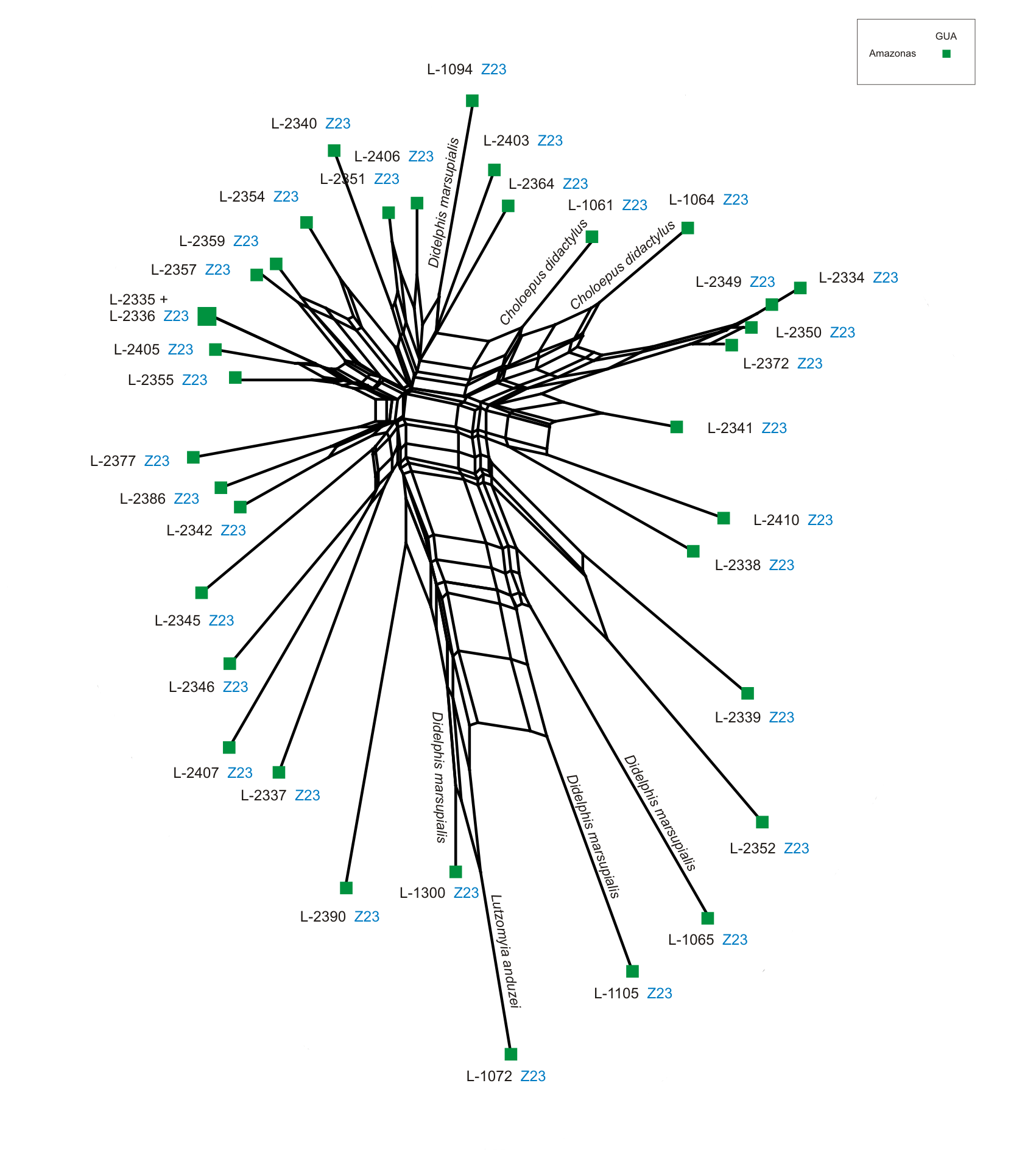

Supplement: Figure S3 — NeighborNet network of Population 1 as inferred by STRUCTURE. Six strains were isolated from animal hosts, two from Choloepus didactylus and four from Didelphis marsupialis, and one from a sand fly, Lutzomyia anduzei, all other strains were isolated from human CL cases. (TIF) [file pntd.0002490.s003.tif]

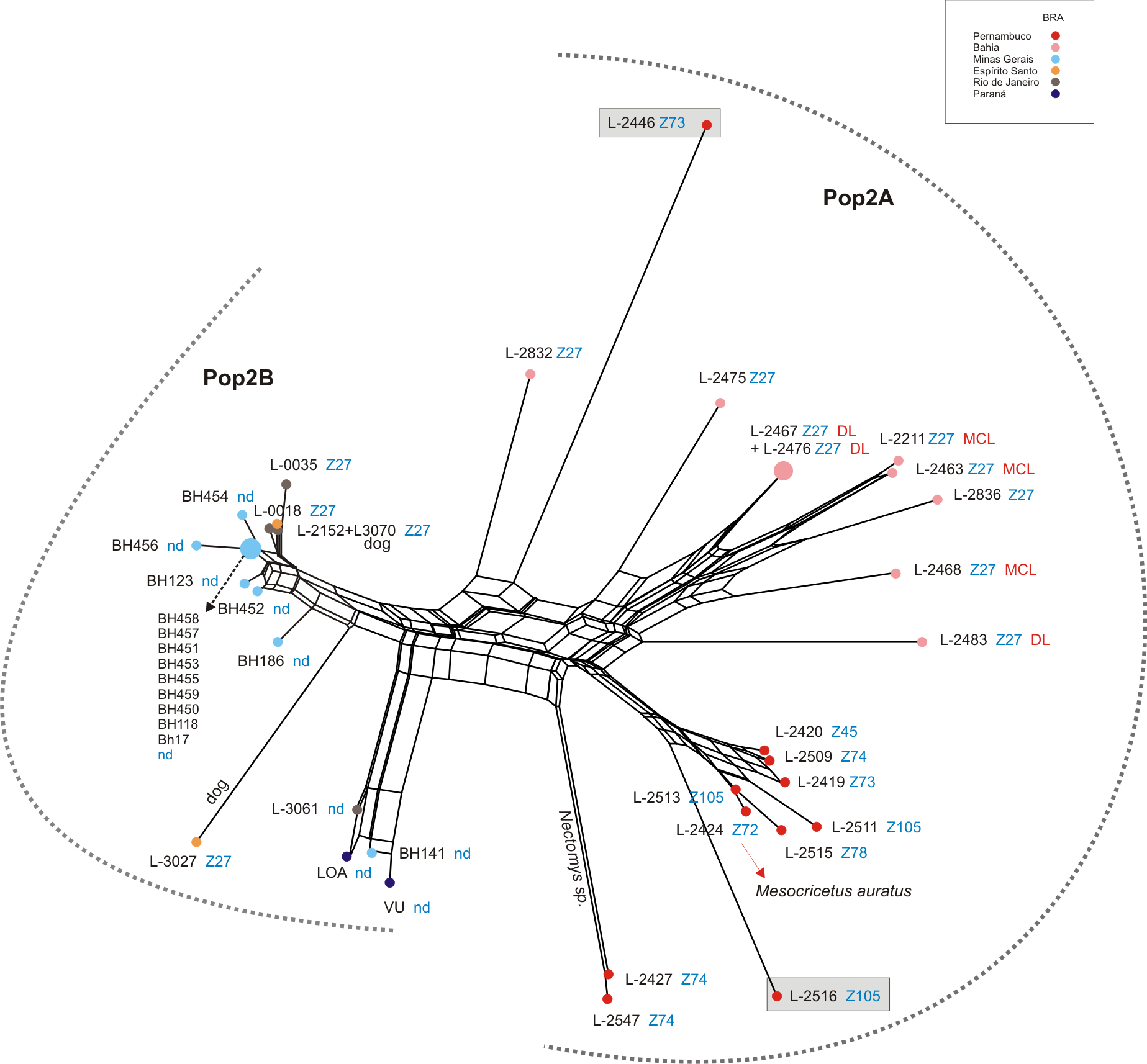

Supplement: Figure S4 — NeighborNet network of Population 2 as inferred by STRUCTURE. Four strains were isolated from animal hosts, two from dogs and one each from Nectomys sp. and Mesocricetus auratus, three from human MCL and three from human DL cases, all other strains were isolated from human CL cases. The assignment of the strains to the two sub-populations of Population 2, A and B, is indicated. Strains presenting mixed membership coefficients in two sub-populations are highlighted in grey. The two sub-populations are largely confirmed by the phylogenetic network albeit some strains occur at intermediate positions. (TIF) [file pntd.0002490.s004.tif]

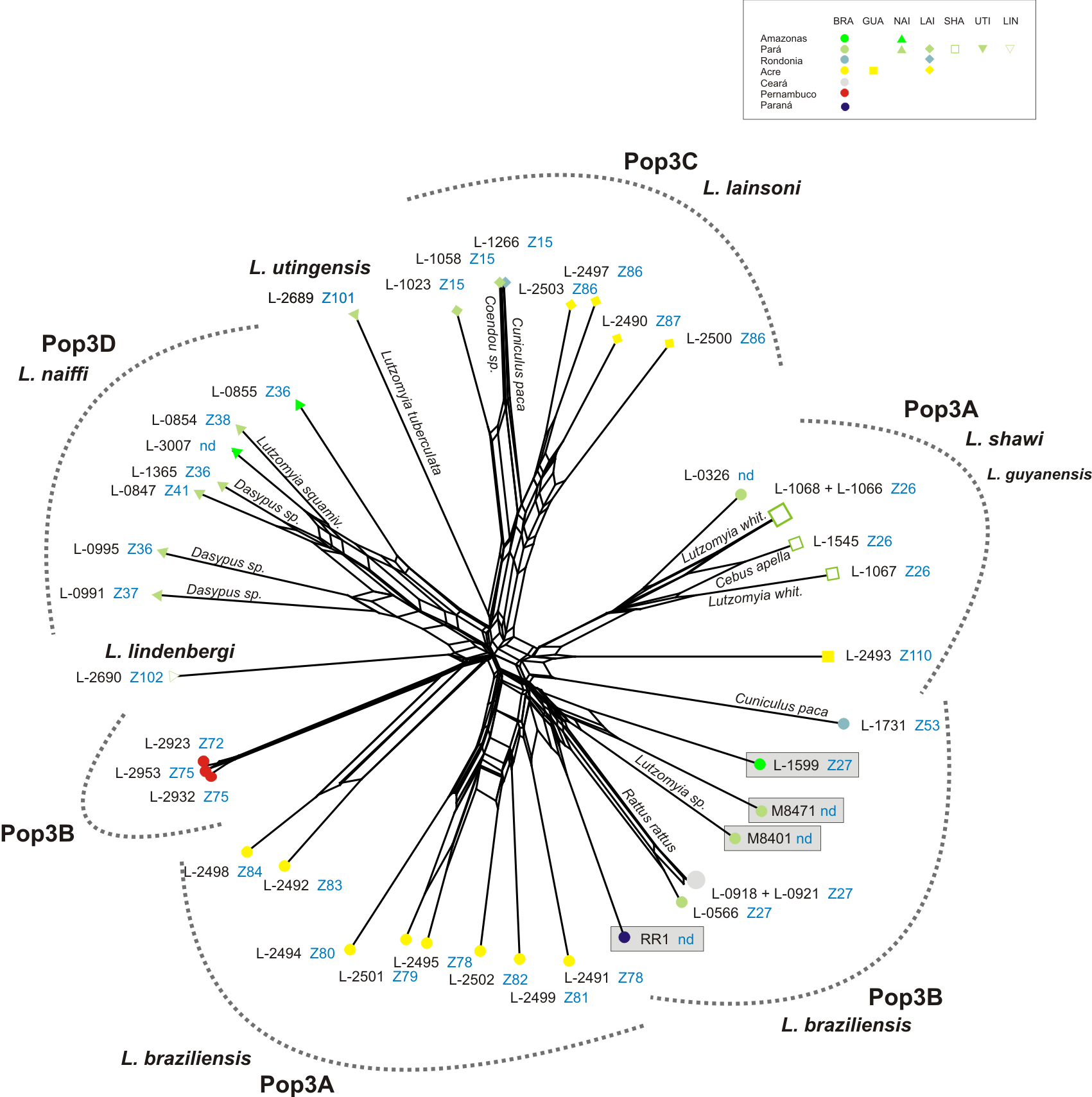

Supplement: Figure S5 — NeighborNet network of Population 3 as inferred by STRUCTURE. Eight strains were isolated from animal hosts, three from Dasypus sp., two from Cuniculus paca and one each from Coendou sp., Cebus apella and Rattus rattus, five from sand flies, three from Lutzomyia whitmani and one each from L. tuberculata, L. squamiventris and Lutzomyia sp., all other strains were isolated from human CL cases. The four sub-populations of Population 3, A, B, C and D, are indicated. The phylogenetic network confirms the assignment of strains of L. (V.) lainsoni to sub-population C and that of strains of L. (V.) naiffi to sub-population D. The sub-populations A and B are not well supported in this NeighborNet network. Strains of L. (V.) shawi were found on a separate branch together with one strain of L. (V.) braziliensis from Pará, L-0326, and the single strain of L. (V.) guyanensis from Acre, L-2493. (TIF) [file pntd.0002490.s005.tif]

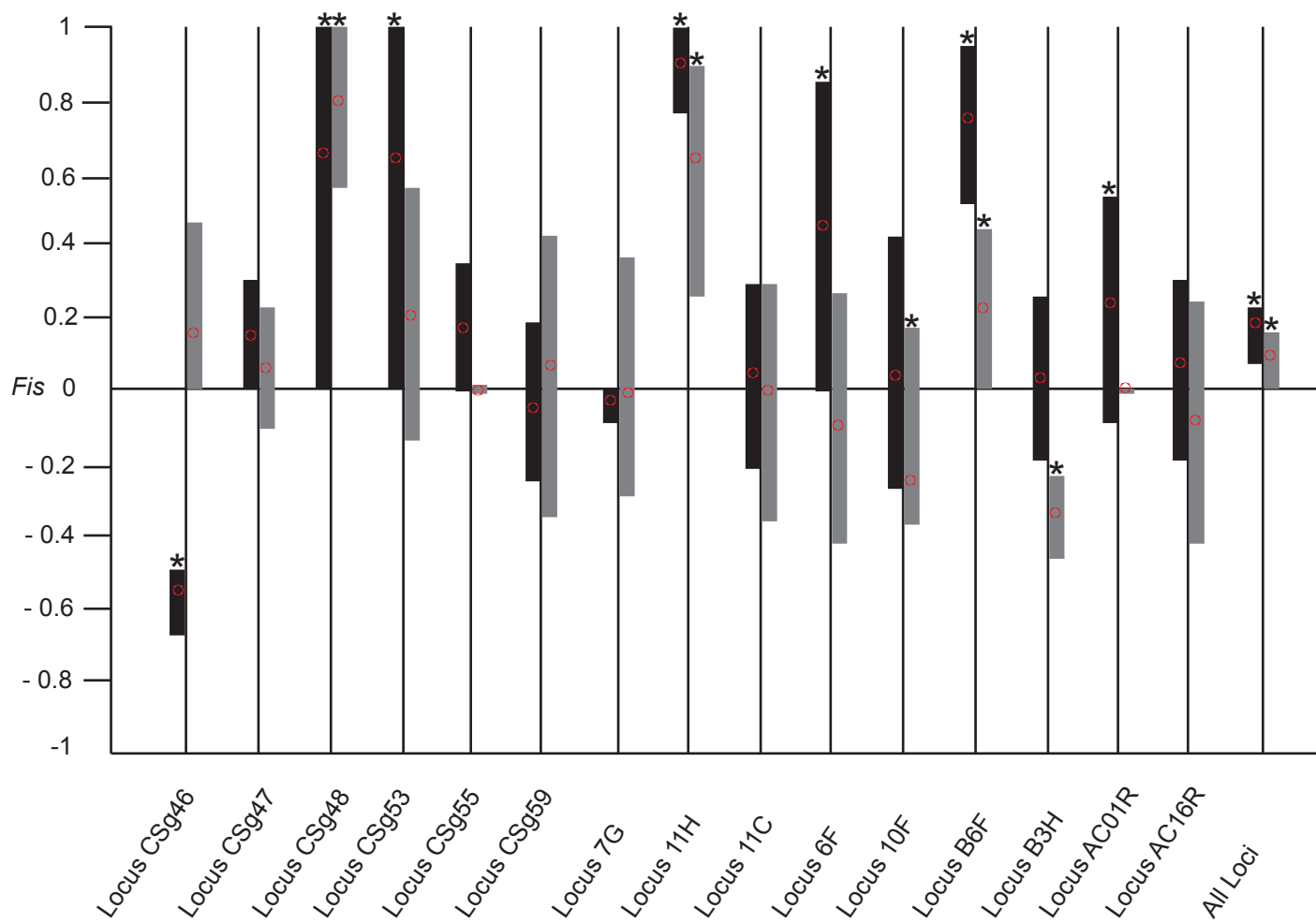

Supplement: Figure S6 — Calculation of inbreeding coefficients for Populations 1 and 2. Fis values were calculated for each of the 15 loci and over all loci. Strains with identical genotypes were excluded from the analyses to avoid medical driven sampling bias and clones over-representation. For each locus, 95% confidence intervals (CI) were obtained by bootstrapping over loci (GENETIX). P values are indicated by stars (P<0.05). (PDF) [file pntd.0002490.s006.pdf]
