# Supplementary material for: Population Structure and Evidence for Both Clonality and Recombination among Brazilian Strains of the Subgenus Leishmania (Viannia)
Source: PLoS Negl Trop Dis. 2013 Oct 31;7(10):e2490. doi: 10.1371/journal.pntd.0002490 (PMC3814519; doi:10.1371/journal.pntd.0002490)
Supplement: Table S2 — Distribution of zymodemes and animal hosts for the populations and sub-populations found by MLMT. (DOCX) [file pntd.0002490.s008.docx]

**Supplementary Information: Table S2**

| **Subpopulation** | **Species** | **Origin** | **Zymodemes [N]** | **Host/vector** |
| --- | --- | --- | --- | --- |
| Pop1 | *L. guyanensis* | Amazonas [36/36] | Z23 [36/36] | human  *Choloepus didactylus* [2/2]  *Didelphis marsupialis* [4/4]  *Lutzomyia anduzei* [1/1] |
| subPop2A | *L. braziliensis* | Bahia [9/9]  Pernambuco [11/14]  Espírito Santo [1/2] | Z27 [9/18]  Z45 [1/1]  Z72 [1/2]  Z73 [2/2]  Z74 [3/3]  Z78 [1/3]  Z105 [3/3]  Z27 [1/18] | human  human  human  *Mesocricetus auratus* [1/1]  human  human  *Nectomys sp.* [1/1]  human  human  canine [1/2] |
| subPop2B | *L. braziliensis* | Minas Gerais [15/15]  Rio de Janeiro [4/4]  Espírito Santo [1/2]  Paraná [2/3] | nd  Z27 [3/18] , nd  Z27 [1/18]  nd | human  human  canine [1/2]  human |
| subPop3A | *L. braziliensis*  *L. shawi*  *L. guyanensis*  *L. utingensis*  *L. lindenbergi* | Acre [6/8]  Rondonia [1/1]  Pará [1/4]  Pará [4/4]  Acre [1/1]  Pará [1/1]  Pará [1/1] | Z78 [2/3]  Z79 [1/1]  Z80 [1/1]  Z81 [1/1]  Z82 [1/1]  Z53 [1/1]  nd  Z26 [4/4]  Z110 [1/1]  Z101 [1/1]  Z102 [1/1] | human  human  human  human  human  *Cuniculus paca* [1/2]  human  *Cebus apella* [1/1]  *Lutzomyia whitmani* [3/3]  human  *Lutzomyia tuberculata* [1/1]  human |
| subPop3B | *L. braziliensis* | Pará [3/4]  Amazonas [1/1]  Paraná [1/3]  Ceará [2/2]  Pernambuco [3/14] | Z27 [1/18], nd  Z27 [1/18]  nd  Z27 [2/18]  Z72 [1/2]  Z75 [2/2] | human  *Lutzomyia sp.* [1/1]  human  human  human  *Rattus rattus* [1/1]  human  human |
| subPop3C | *L. lainsoni* | Acre [4/4]  Pará [2/2]  Rondonia [1/1] | Z86 [3/3],  Z87 [1/1]  Z15 [2/3]  Z15 [1/3] | human  human  human  *Cuniculus paca* [1/2]  *Coendou spec.* [1/1] |
| subPop3D | *L. naiffi*  *L. braziliensis* | Pará [2 /5]  [1/5]  [1/5]  [1/5]  Amazonas [2/2]  Acre [2/8] | Z36 [2/3]  Z37 [1/1]  Z38 [1/1]  Z41 [1/1]  Z36 [1/3]  Z83 [1/1]  Z84 [1/1] | human  *Dasypus sp.* [2/3]  *Dasypus sp.* [1/3]  *Lutzomyia squamiventris* [1/1]  human  human  human  human |

Zymodemes occurring in different subpopulations are marked in red, blue or green color, respectively.
